# Highly Sensitive Detection of 4-Methylimidazole Using a Terahertz Metamaterial

**DOI:** 10.3390/s18124304

**Published:** 2018-12-06

**Authors:** Hee Jun Shin, Hae Won Jang, Gyeongsik Ok

**Affiliations:** 1 Pohang Accelerator Laboratory, 80 Jigokro-127-beongil, Nam-Gu, Pohang, Gyeongbuk 37673, Korea; shinhj@postech.ac.kr; 2 Research Group of Food Processing, Korea Food Research Institute, 245, Nongsaengmyeong-ro, Iseo-myeon, Wanju-gun, Jeollabuk-do 55365, Korea; hwjkfri@kfri.re.kr; 3 Research Group of Consumer Safety, Korea Food Research Institute, 245, Nongsaengmyeong-ro, Iseo-myeon, Wanju-gun, Jeollabuk-do 55365, Korea

**Keywords:** THz spectroscopy, 4-methylimidazole, metamaterial, carcinogen detection

## Abstract

In this study, we demonstrated a highly sensitive detection method of 4-methylimidazole (4-MeI), a carcinogenic material, by using a terahertz (THz) metamaterial at a THz region. The THz metamaterials were fabricated with a metal array, using an electric-field-coupled inductor-capacitor (ELC) resonator structure, and a finite-difference time-domain (FDTD) simulation showed good agreement with the experimental results. We measured the THz spectra of the metamaterials to detect the 4-MeI concentrations of 0, 1, 2, 5, 10, 15, and 20 mg/L. The resonance frequency of the metamaterial was shifted by, approximately, 8 GHz and transmittance at the resonance frequency increased to 2 × 10^−3^, as the concentration was increased, up to 20 mg/L. Our study provides new insight into the application of metamaterials in detecting carcinogens, using a THz technique.

## 1. Introduction

Several soft drinks, including sodas, energy drinks, and diet drinks, contain a caramel color which is widely used as a coloring and flavoring agent [1,2]. Caramel-colored drinks, including colas, root beer, and black tea, are widely consumed across the world in large amounts. However, caramel color agents have attracted significant attention because it was found that caramel-colored drinks may contain 4-methylimidazole (4-MeI), which is known to be carcinogenic [1,3,4,5]. Caramel colors are usually classified into four types, Caramel I, II, III, and IV, and their production and choice of reagents are based on their applications [2,3]. The compound 4-MeI is formed during the production of caramel colors type III and IV, which contain ammonium compounds. Detecting 4-MeI in food products has been an ongoing challenge, with several studies directed at developing efficient methods. For example, high performance liquid chromatography (HPLC) [6], HPLC-mass spectroscopy [3,7], gas chromatography (GC) [8], and GC-mass spectroscopy [9,10], have been used to detect 4-MeI, in addition to 2-dimensional liquid chromatography [11,12]. Spectral properties of various imidazole compounds and characteristics of imidazole isomers can be investigated using nuclear magnetic resonance and mass spectroscopy [13,14]. However, new detection techniques using label-free technologies, with high sensitivity and ease of use, have been in high demand, especially, in cases of chemical or biological material testing.

Following recent reports that terahertz (THz) waves can be generated using a femtosecond laser, application of the THz frequency range has gained significant attention in various research areas, including biochemistry [15,16,17,18], electronics [19], photonics [20,21], astronomy [22], military science [23], food science [24,25], and medical diagnosis [26,27]. In particular, THz techniques such as THz time-domain spectroscopy, have emerged as attractive techniques that can enable label-free, non-contact, and non-destructive detection in biological and chemical materials [18,28,29]. The THz range can reveal several types of information in biological and chemical materials; for example, various conformational energies, such as rotation, vibration, and intermolecular interactions of chemical and biological molecules occur in the THz range [18,24,30]. Recent efforts to improve sensitivity of biochemical detection have involved the use of various artificial structures that are fabricated using metamaterials. Metamaterials have several significant advantages, including easy preparation, easy handling, high sensitivity, and material-selective procedures. Usually, artificial structures created using conductive materials, such as metals, are used for patterning metamaterials, and this provides an opportunity to control electromagnetic properties and functionalities of the materials. In addition, metamaterials have been designed to function in various frequency ranges, using their unique properties, including negative refractive index, and high absorbance at specific frequencies. In particular, THz metamaterials have been widely studied because they can be used to design artificial structures to enhance the detection of several chemical and biological materials in the THz range. THz metamaterials can be used to markedly enhance biochemical molecular energies in the THz region, and they have been investigated in various biological and chemical studies [31,32,33,34,35,36,37,38,39].

In the present study, we demonstrated the detection of 4-MeI, a possible carcinogenic component in caramel-colored drinks, using THz metamaterials. We measured the THz spectrum of 4-MeI, in the frequency range of 0.2 to 2 THz, using the THz transmission mode spectroscopy. Using a THz metamaterial at the resonance frequency of 4-MeI, we investigated its frequency shift enhancement for 4-MeI detection.

## 2. Materials and Experiment

### 2.1. Metamaterial Fabrication

**Figure 1:**
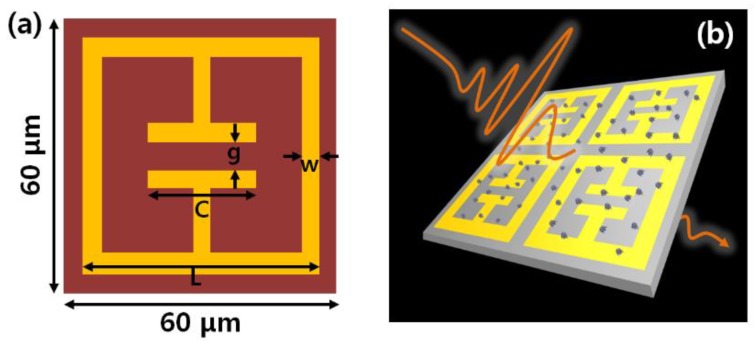
Schematic of (**a**) the terahertz (THz) metamaterial and (**b**) THz measurement, using the metamaterial.

The THz metamaterial was fabricated using an electric-field-coupled inductor-capacitor (ELC) resonator structure [40,41]. The metamaterial was based on a metallic array consisting of a square ring with a micro split gap structure. A polyimide (PI) solution was spin-coated onto the Si wafer, at a thickness of 500 μm, at 2500 rpm. The Si wafer with a coated PI solution was baked at 110 °C, for 3 min. To deposit an Au pattern on the PI/Si wafer, we adopted a lift-off process. The PI/Si wafer substrate was photoresist-coated at 3000 rpm for 40 s and soft baked at 95 °C for 90 s. Next, the substrate was exposed to UV for three seconds. Hard baking was performed at 110 °C, for 3 min, followed again by UV exposure for 30 s. Finally, the photoresist was developed. We deposited a Ti 30 nm/Au 100 nm layer on the PI/Si wafer. The schematic of the metamaterial is shown in Figure 1a. We simulated the metamaterial design using a finite-difference time-domain (FDTD) method, in the terahertz range.

As can be seen in Figure 1b, a THz electric field passing through the metamaterial was scattered by the 4-MeI molecules in a vertical direction. The THz output signal throughout the metamaterial, was analyzed using a Fourier transformation-based method. A commercial THz time-domain spectroscopy system (TPS-3000, Teraview, Cambridge, UK) was used to obtain the THz spectra of the pellet sample and the metamaterial, in the frequency range of 0.2 to 2 THz. All of the experimental THz-TDS measurements were performed at room temperature and the humidity in the THz-TDS system was maintained at lower than 1%, using dried air, during the THz spectra measurement of the samples.

The output THz signal *O*(*ω*), passing through a sample, is related to the input signal *I*(*ω*) as follows [18]

(1) $$O\left(\omega \right)=I\left(\omega \right)\exp\left[-\frac{d\alpha \left(\omega \right)}{2}\right]\exp\left[i\frac{2\pi }{\lambda }n_{1}\left(\omega \right)d\right],$$

where *I*(*ω*) is the complex input signal, *α*(*ω*) is the absorption coefficient, *d* is the thickness of the sample, *λ* is the wavelength, and *n* is the refractive index of the sample. Power absorption can be obtained from the difference between spectral amplitudes with and without the sample.

(2) $$\alpha \left(\omega \right)=-\frac{2}{d}ln\left[\frac{O\left(\omega \right)}{I\left(\omega \right)}\right].$$

### 2.2. 4-MeI Sample Preparation

**Figure 2:**
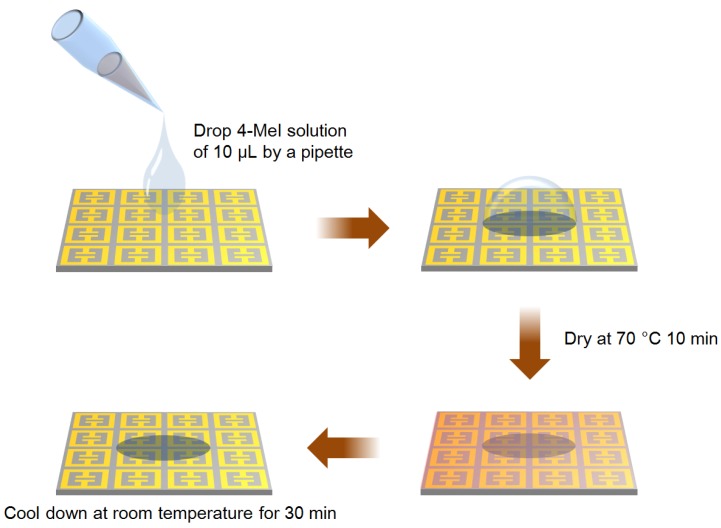
Schematic of the drop coating deposition of the 4-methylimidazole (4-MeI) sample on the THz metamaterial.

A commercially available 4-MeI product was obtained (Sigma-Aldrich, St. Louis, MO, USA). We crushed the dry 4-MeI into small particles of sizes less than the THz wavelength, to prevent scattering. To measure the THz spectrum, the 4-MeI (30 mg) was prepared as a pellet sample compounded with a polyethylene (PE) powder (270 mg). In addition, we prepared 4-MeI solutions to apply onto the THz metamaterial, at concentrations of 0, 1, 2, 5, 10, 15, and 20 mg/L of 4-MeI, in distilled water. Ten microliter of the 4-MeI solutions, at each concentration, were dropped onto the THz metamaterial and dried at 70 °C, for 10 min, until the water evaporated completely. Next, the THz metamaterial was cooled, at room temperature, for 30 min to maintain identical conditions for each measurement. A schematic of the process is shown in Figure 2.

## 3. Results and Discussion

### 3.1. THz Spectrum of a 4-MeI Pellet

**Figure 3:**
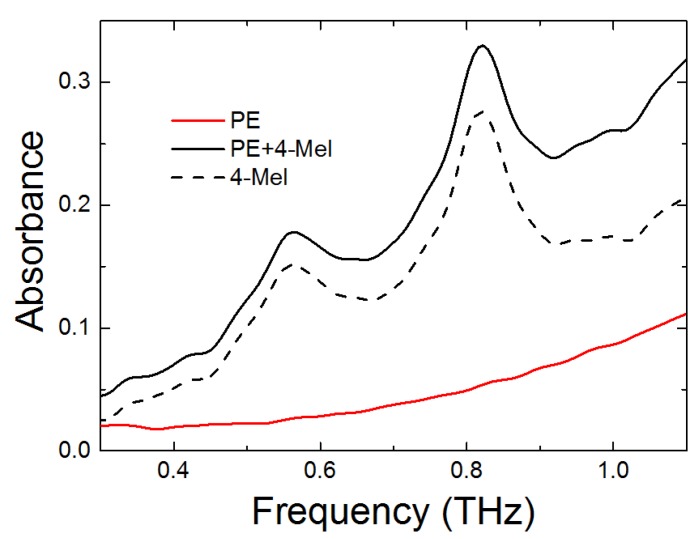
The spectra of polyethylene and 4-methylimidazole pellets at the THz region of 0.2 to 1.1 THz.

We first described the THz spectral characteristics of the 4-MeI, using THz-TDS. We measured the THz spectra of a pellet composed of PE and 4-MeI, and the spectra are shown in Figure 3. As can be seen in the figure, the PE pellet showed a low absorption and had no resonance peak at the measured THz region. From the THz spectrum of the PE pellet, we obtained the THz spectrum of 4-MeI, by extracting the absorbance of the PE. The spectrum of the pellet composed of PE and the 4-MeI showed two significant 4-MeI resonance peaks at 0.56 and 0.82 THz. In particular, the spectrum of 4-MeI showed a more intense absorption peak at 0.82 THz than at 0.56 THz.

### 3.2. 4-MeI Detection in the THz Metamaterial

**Figure 4:**
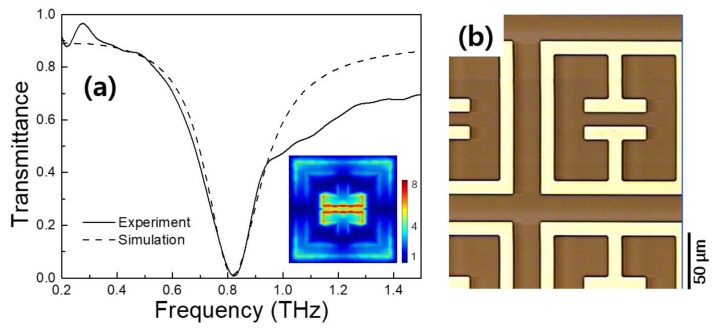
(**a**) Transmittance curves obtained in the experiment and the simulation, using the THz metamaterial. (**b**) Optical microscope image of the THz metamaterial.

To fabricate frequency-selective metamaterials for 4-MeI, as shown in Figure 1, the metamaterial was designed to encompass a resonance frequency of 0.82 THz, which was the resonance frequency of the 4-MeI. The resonant peak position of the transmittance was determined, based on the capacitor length and the gap of the metamaterial [31]. The capacitor length (C) and the gap (g) were designed to be 20 μm and 8 μm. The full length (L) of the metamaterial was 54 μm, as shown in Figure 1a. The simulation and experimental results are shown in Figure 4a. In the FDTD simulation, gold is considered to be a perfect electric conductor (PEC) and the relative permittivity of the PI layer was 2.88-0.09i. The normalized THz spectrum of the metamaterial illustrated a resonant feature at 0.82 THz. As can be seen in Figure 4a, the experimental spectrum of the THz metamaterial was in good agreement with the FDTD simulation. In addition, at the resonance frequency, we found that the localized THz electric field was strongly enhanced at the gap of the antenna, in the simulation, as shown in the inset, in Figure 4a. Figure 4b shows a scanning optical microscope image of the fabricated THz metamaterial. When the 4-MeI sample was dropped onto the THz metamaterial, the 4-MeI molecules significantly interacted with the localized strong THz field and the features of the metamaterial changed as the 4-MeI concentration increased. Consequently, the resonant peak position and the transmittance were changed.

**Figure 5:**
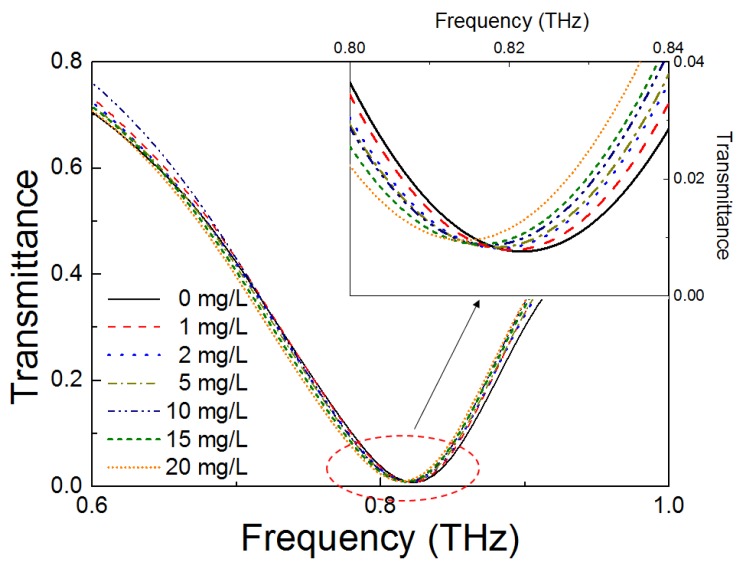
Spectra of the 4-MeI detection, using the THz metamaterial, at concentrations of 0, 1, 2, 5, 10, 15, and 20 mg/L in the THz region of 0.6 to 1.0 THz.

**Figure 6:**
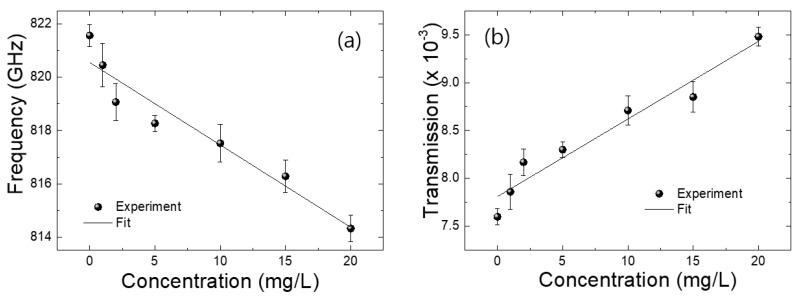
(**a**) The resonance frequency and (**b**) transmittance of the THz metamaterial as the concentration of 4-MeI increases. Solid lines indicate linear fits of the experimental results. All experimental results have been obtained through four different times of measurement.

Figure 5 illustrates the full THz spectra of the metamaterial, at different 4-MeI concentrations of 0, 1, 2, 5, 10, 15, and 20 mg/L. The inset in Figure 5 shows a zoomed-in image of the resonant region. As seen in the figure, the resonant peak of the THz metamaterial shifted to a lower frequency as the 4-MeI concentration increased. In addition, the transmittance also increased slightly. The 4-MeI concentration-dependence of the resonance frequency and the transmittance of the THz metamaterial is shown in Figure 6. In Figure 6a, the resonance frequency was shifted to a lower frequency as the concentration of the 4-MeI increased. In addition, the transmittance increased as the concentration of the 4-MeI increased. The behavior of the resonance frequency and the transmittance could be explained by fitting to a linear equation. The resonance frequency shift-fitted to the linear equation Y = −0.31X + 820.56 and the transmittance change-fitted to Y = 8.1 × 10^−3^X + 7.8 × 10^3^, with increasing 4-MeI concentrations. As a result, the resonance frequency was shifted, by approximately 8 GHz, and the transmittance at the resonance frequency increased to 2 × 10^−3^ as the concentration increased to 20 mg/L. These results indicated that a few mg/L of the 4-MeI present in food materials, such as a beverage, can be detected using this metamaterial. However, the sensitivity of the metamaterial needs to be improved by using a higher sharp resonant peak or enhancing the interactions between the over-layered materials and an electric field near the metal structure, which would enable detection of 4-MeI of less than a few hundred mg/L.

The present study provided experimental evidence supporting the fact that the application of metamaterials, combined with the THz technology, has a substantial potential in the detection of hazardous materials in food products. Further studies may involve fabrication of the THz metamaterials with sharp resonant peaks and a high sensitivity, with a nano-slit structure, to improve the sensitivity of the metamaterials in detecting 4-MeI, as well as other carcinogenic substances.

## 4. Conclusions

In conclusion, we reported a THz metamaterial-based detection of the carcinogenic substance 4-MeI, which may be present in caramel-colored food products. We measured the THz spectra of pellets containing 4-MeI and PE and observed the resonance peaks at 0.56 and 0.82 THz. We fabricated a THz metamaterial, with a resonance feature at 0.82 THz, and measured the THz spectra at 4-MeI concentrations ranging from 0 to 20 mg/L. We found that the resonance frequency decreased and the transmittance increased as the 4-MeI concentration increased. We were able to clearly detect 4-MeI, using the THz metamaterial, and the results demonstrated a highly sensitive detection. Thus, our findings provide new insight into 4-MeI detection and the potential applications of metamaterials-based technology, in food safety inspection.
